# A Decade of Innovation: Short-Term Outcomes of 150 Robotic Liver Resections

**DOI:** 10.3390/jcm14186530

**Published:** 2025-09-17

**Authors:** Alessio Pasquale, Francesco A. Ciarleglio, Laura Marinelli, Giovanni Viel, Stefano Valcanover, Nick Salimian, Stefano Marcucci, Marco Brolese, Paolo Beltempo, Alberto Brolese

**Affiliations:** 1 Department of General Surgery, Hepato-Pancreato-Biliary (HPB) Unit—Azienda Provinciale per i Servizi Sanitari APSS, 38121 Trento, Italy; 2 Department of General Surgery—Azienda Provinciale per i Servizi Sanitari APSS, 38023 Cles, Italy; 3 Department of Surgery, Oncology and Gastroenterology, Hepatobiliary Surgery and Liver Transplantation, Padova University, 35100 Padova, Italy

**Keywords:** hepatobiliary and pancreatic surgery, robotic liver resection, laparoscopic liver resection, mini invasive liver surgery, liver tumors

## Abstract

**Background:** Robotic liver resection (RLR) has seen remarkable advancements in recent years, overcoming many limitations of laparoscopic liver resection (LLR). RLR has evolved to include increasingly complex procedures, offering enhanced precision, reduced blood loss, and lower complication rates. **Materials and Methods:** A total of 150 consecutive RLRs, performed at the Department of General Surgery II and HPB Unit of Santa Chiara Hospital (Trento, Italy), between January 2013 and June 2024 were retrospectively reviewed. Collected data included demographics, disease etiology, operative parameters, oncologic margins, and perioperative outcomes. **Results:** Indications were malignant disease in 83% of cases while benign disease accounted for 17%. Minor resections accounted for 91%. Cirrhosis was present in 49% of patients (Child–Pugh A 91%; B 9%; mean MELD 9). According to the Iwate difficulty score, resections were low difficulty in 38% of cases, intermediate in 50%, advanced in 7%, expert in 5%. Conversion rate was 12%, mainly for bleeding or adhesions. Mean blood loss was 159 mL (66% <100 mL); Pringle maneuver was used in 3%; drains omitted in 45%; ICG fluorescence used in 81%. Mean operative time was 250 min (console time 184 min). Mean lesion size was 34 mm; R0 margin rate was 82%. Overall mortality was 1.3%; morbidity 24% (Clavien–Dindo ≥ III in 10%). Mean hospital stay was 7 days (median 5; range 2–46). **Conclusions:** RLR is a safe and effective alternative to laparoscopy, providing comparable or superior perioperative outcomes. Medium-volume centers can achieve high-quality results with RLR. Continued technological advancements will further expand its applications to increasingly complex liver procedures.

## 1. Introduction

Since the first laparoscopic liver resection (LLR) in the early 1990s [1], minimally invasive liver surgery (MILS) has evolved considerably. In this context, robotic liver resection (RLR) has emerged as a major advancement, with its initial application in hepatobiliary surgery dating back to the mid-1990s, when Himpens et al. performed the first robotic cholecystectomy [2]. However, the introduction of the Da Vinci^®^ robotic platform in the early 2000s marked a pivotal turning point, facilitating broader adoption and representing a breakthrough in the field of MILS.

In 2003, Giulianotti et al. published the first series of robotic liver resections [3], showing that the superior operative control provided by the robotic approach, even in deep-seated and anatomically complex liver segments, helped overcome many of the intrinsic limitations of conventional laparoscopy. Although early diffusion of RLR was limited primarily by high costs, accumulating evidence in the literature has progressively driven its adoption, and an increasing number of centers worldwide are now incorporating RLR into their routine surgical practice [4]. RLR is currently employed in various hepatic diseases, both benign and malignant, including hepatocellular carcinoma (HCC) and colorectal liver metastases (CRLM) [5,6], and more recently, its use has been extended to complex cases of perihilar cholangiocarcinoma [7]. Furthermore, selected high-volume centers have adopted RLR for advanced procedures such as living donor hepatectomy and Associating Liver Partition and Portal vein ligation for Staged hepatectomy (ALPSS) [8,9]. In these demanding scenarios, the robotic platform offers significant technical advantages, such as enhanced dexterity, tremor filtration, and high-definition three-dimensional visualization, that enable precise dissection and suturing, particularly in anatomically challenging regions.

In 2018, the first international consensus conference on robotic liver surgery established structured recommendations based on available evidence and expert consensus [10]. As the field continued to evolve, the Iwate difficulty scoring system was introduced to guide case selection and improve the safety profile of MILS [11]. Finally, RLR has shown excellent perioperative and oncologic outcomes [12] representing a valuable option for hepatobiliary surgeons. The aim of our study is to demonstrate that, with adequate expertise and proper patient selection, this approach can be performed safely and effectively even in medium-volume centers.

## 2. Materials and Methods

We performed a retrospective analysis of 150 consecutive robotic liver resections (RLRs) at the Hepato-Pancreato-Biliary Unit of APSS in Trento, Italy, between January 2013 and June 2024. The average number of hepatic resections (both open and minimally invasive) performed in our Unit is 40/year. All patients were adults (≥18 years) with liver lesions—benign or malignant—diagnosed through CT or MRI, and confirmed by histopathology when indicated. Before surgery, every case was reviewed by our multidisciplinary tumor board to ensure appropriate indications.

Ethical approval was obtained from the local committee, and informed consent was signed by all patients. Procedures were carried out using the Da Vinci^®^ robotic platform: the Si^®^ model was used from 2013 to 2018, and the Xi^®^ model thereafter.

We collected detailed data on patient demographics (including age, sex, BMI, comorbidities, prior abdominal operations, ASA score [13], and liver function assessed via Child–Pugh and MELD), tumor characteristics (type, number, location, size, histology, and serum tumor markers AFP, CEA, CA19-9), intraoperative parameters: operative time (from skin incision to closure), console time (duration on the robotic console), estimated blood loss, transfusion requirements, conversions to open surgery, and Pringle maneuver use), and postoperative outcomes (complications graded by Clavien–Dindo [14] and the Comprehensive Complication Index [15], reoperations, length of hospital stay, and 30-day readmission rate). We did not exclude patients based on prior surgery, large tumor size, vascular involvement, or the need for complex reconstructions, although no vascular reconstructions were performed in this cohort.

All surgeries were led by our chief surgeon, who has over 30 years of experience in liver surgery and solid training in open and laparoscopic techniques. In the program’s early phase, we focused on minor resections—such as wedge resections and anterior segmentectomies—but gradually expanded to resections of posterior-lateral segments and major hepatectomies, including right and left lobectomies and complex Klatskin tumor cases [7].

All patients were managed within Enhanced Recovery After Surgery (ERAS) protocol. Patients were generally positioned supine with a 30° reverse Trendelenburg tilt. For resection involving posterior segment (segments 6 and 7), we used a left lateral decubitus position with a 30–45° tilt. Pneumoperitoneum was established via Veress needle or open technique, at the planned site of the 12 mm AirSeal^®^ trocar, which was then inserted under direct vision, followed by insertion of three to five 8-mm robotic trocars under direct vision. In patients with prior abdominal surgery, initial adhesiolysis was performed laparoscopically with cold scissors and blunt dissection before docking the robot.

Once docked, we systematically explored the abdominal cavity and mobilized the hemiliver under intraoperative ultrasound guidance. The Pringle maneuver was prepared in all cases—initially using an external tourniquet with an Argyle^®^ tube and later an intracorporeal Foley catheter for intermittent hepatic pedicle clamping [16]. Parenchymal transection was performed in a kellyclasia fashion with bipolar and monopolar energy devices, supplemented by a Vessel Sealer^®^ when additional hemostasis was required. Indocyanine green fluorescence imaging (15 mg IV, given either intraoperatively or 48 h before surgery) supported lesion visualization, segmental mapping, and vascular assessment [17].

Vascular and biliary structures were divided using Hem-o-Lok^®^ clips or staplers, and specimens were retrieved in a laparoscopic extraction bag through either an enlarged trocar site or a small Pfannenstiel incision. This standardized approach ensured consistency, safety, and reproducibility across our series of robotic liver resections.

### Statistical Analysis

Demographic data and clinical outcomes were analyzed statistically. Descriptive analysis included the mean and standard deviation for continuous variables, while categorical and ordinal variables were presented as counts and proportions. A *p*-value < 0.05 was considered statistically significant. Statistical analyses were conducted using SPSS Statistics version 22.0 (IBM, Armonk, NY, USA).

## 3. Results

**Figure 1:**
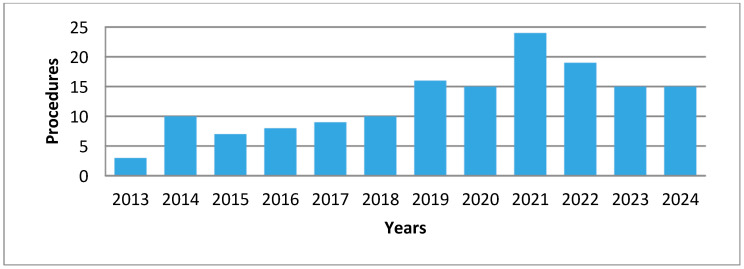
Number of procedures *N* = 150.

Between January 2013 and June 2024, a total of 150 robotic liver resections (RLRs) were performed at the Department of General Surgery and Hepato-Pancreato-Biliary (HPB) Unit, APSS, Trento, Italy. The number of procedures increased steadily over time, reaching a peak in 2019. A temporary decline occurred in 2020, likely due to the COVID-19 pandemic and its impact on elective surgical activity. Volumes subsequently recovered in the following years (Figure 1).

The median patient age was 68 years (range 21–86), with a mean BMI of 26 ± 4 kg/m^2^. Among the cohort, 32% of patients were classified as ASA III–IV, 38% had a history of previous abdominal surgery, 15% had diabetes mellitus, 36% hypertension, and 25% had cardiac comorbidities. Liver cirrhosis was present in 74 patients (49.3%), of whom 91% were Child–Pugh class A and 9% class B (range A5–B7), with a mean MELD score of 9 (range 6–18).

83% of resections were performed for malignant tumors. Hepatocellular carcinoma (HCC) and colorectal liver metastases (CRLM) were the most common indications, accounting for 48% and 21% of cases, respectively. Cholangiocarcinoma represented 4% of indications, gallbladder carcinoma 1%, and other non-CRLM metastases 9%.

In 83% of patients, resection was performed for a single lesion. The mean lesion size was 34 mm (range 10–150 mm), and the R0 resection rate was 82%. Minor resections represented 91.5% of cases and included 62% wedge resections, 15.5% anatomical segmentectomies, and 14% bisegmentectomies. Major resections (defined as resection of ≥3 contiguous segments) comprised 8.5% of procedures: 8 left hepatectomies, 4 right hepatectomies, and 1 central hepatectomy. In two cases, left hepatectomy was combined with segment 1 resection and jejunostomy for hilar cholangiocarcinoma (Klatskin tumors).

Synchronous colorectal and liver resections were performed in seven cases, all involving minor liver resections. These included two anterior rectal resections, three right colectomies, and two left colectomies (Table 1, Table 2 and Table 3).

**Figure 2:**
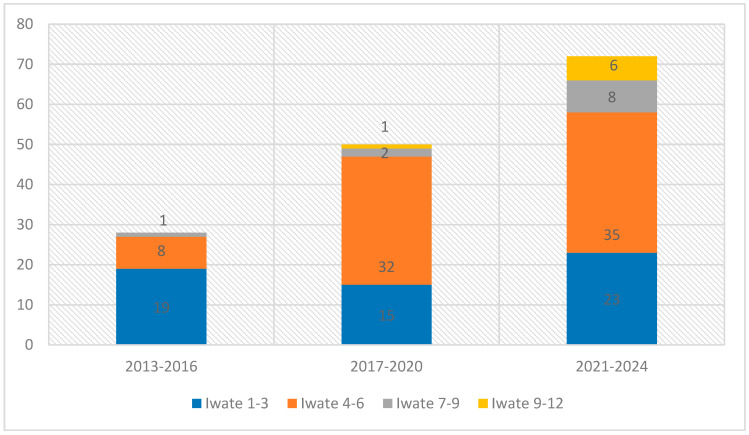
Distribution of Iwate scores over three periods (χ^2^ = 19.6; df = 6; *p* = 0.003).

Resections were classified using the Iwate difficulty score: 38% low (0–3), 50% intermediate (4–6), 7% advanced (7–9), and 5% expert (10–12). When we split the study period into three intervals (2013–2016, 2017–2020, 2021–2024), a clear pattern emerged: early on, simpler cases (Iwate 1–3) were performed robotically, but over time these shifted to laparoscopy, while the robot was increasingly reserved for more complex resections. Intermediate-difficulty cases rose from 8 in the first interval to 32 and then 35, and advanced/expert cases also became more frequent, reflecting growing confidence in the robotic approach. A Pearson χ^2^ test confirmed this trend toward higher-complexity robotic use (χ^2^ = 19.6, df = 6; *p* = 0.003) (Figure 2).

**Figure 3:**
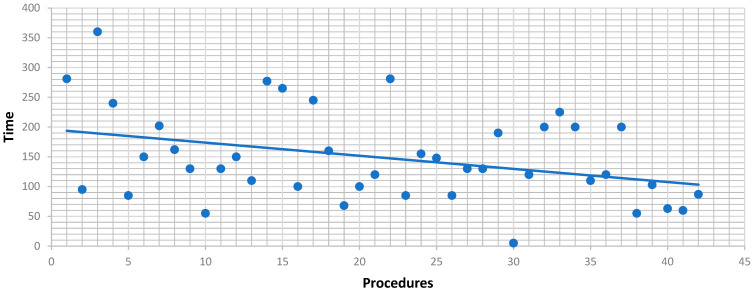
Correlation between number of procedures and console time (Iwate 1–3).

**Figure 4:**
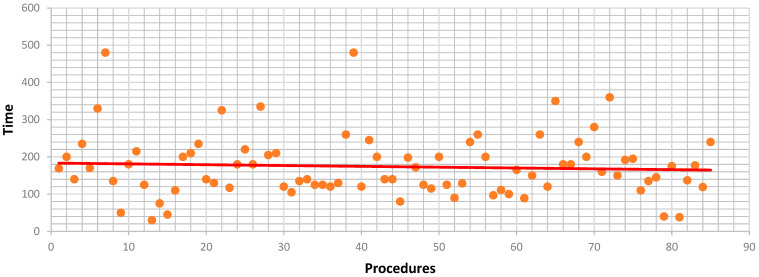
Correlation between number of procedures and console time (Iwate 4–6).

Console time analysis showed that low-difficulty resections had a mean of 148 min (median 130; range 5–360; SD = 76.3). A linear regression indicated a significant reduction in console time with increasing case number (slope = –2.21 min per case, R^2^ = 12.6%, *p* = 0.021; Figure 3). Intermediate-difficulty resections had a mean console time of 174 min (median 160; range 30–480; SD = 84.3), but regression showed no significant reduction (slope = –0.22 min/case; R^2^ = 0.4%, *p* = 0.555) (Figure 3 and Figure 4).

ICG fluorescence was used in 81% of cases, either intraoperatively or 48 h preoperatively, depending on the clinical indication.

The mean operative time was 250 ± 119 min (range 77–840), with a mean console time of 184 min (range 5–700). Conversion to open surgery occurred in 12% of cases (18 patients), primarily due to bleeding or adhesions.

Mean estimated blood loss was 159 ± 228 mL (range 0–2000), with 66% of patients losing < 100 mL. Blood transfusion was required in 5% of cases. The Pringle maneuver was used in only 3%, and abdominal drainage was omitted in 43%.

Postoperative morbidity was 24%, with 10% classified as Clavien-Dindo grade III or higher. Bile leaks occurred in 6% of patients (80% managed conservatively), ascites in 3.3%, and liver failure in 0.5% (1 case). The reoperation rate was 5%, and 30-day readmission was 3%. Overall mortality was 1.3% (2 patients). Median hospital stay was 5 days (range 2–46) (Table 4).

## 4. Discussion

Over the years, the development of MILS has played a pivotal role in the field of hepatic surgery and has become increasingly adopted worldwide. While LLR is widely accepted and established for various indications [18], RLR has emerged as a promising alternative by addressing several limitations of traditional laparoscopy, offering unique advantages such as improved ergonomics, enhanced visualization, and superior instrument articulation, features that are especially beneficial in complex procedures involving deep or posterior liver segments [3].

This study reviews the outcomes of 150 consecutive RLR conducted in our center over the last decade, demonstrating the feasibility and safety of the robotic approach in different fields of liver surgery.

We took a systematic approach to RLR and built a dedicated team that quickly became proficient, speeding up docking times, operational prep, and room setup. Our patient cohort, 66% male with a median BMI of 24 kg/m^2^, closely matching typical Western populations [19]. While over 91% of the procedures performed were minor resections (defined as resection of ≤2 adjacent Couinaud segments), over time we observed a consistent increase in the number of complex robotic liver resections. This trend reflects not only technical maturation but also improved preoperative planning and growing confidence in the robotic approach for advanced cases.

In our case series, blood loss was less than 100 mL in 66% of cases, and the overall conversion rate was 12%, primarily due to bleeding or adhesions. These outcomes are consistent with previously published data [8]. This highlights how reliable our results are and adds to the growing evidence that RLR is safe and feasible.

The majority of cases (85%) were performed for malignant lesions and Indocyanine Green (ICG) was routinely administered for lesion detection, parenchymal demarcation, and vascular assessment, employing both positive and negative staining techniques in conjunction with intraoperative ultrasound [17]. Although nearly half of our patients (49%) had underlying liver disease and most (91%) were Child–Pugh A, a notable number of Child–Pugh B patients also successfully underwent RLR. This shows that, with careful selection, RLR is feasible even in more complex cases. Overall, our findings demonstrate that RLR can be adapted for a wide range of patient conditions, including those with impaired liver function.

In our series, the difficulty of liver resections progressively increased over time, mirroring a marked enhancement in both surgical expertise and technical proficiency. This progression was accompanied by a strategic shift: lower-difficulty resections (Iwate 1–3) have been increasingly managed laparoscopically, while the robotic platform has been selectively employed for more complex cases. By reserving RLR for anatomically demanding procedures, particularly those involving posterior or superior segments, or requiring precise vascular and biliary dissection, we have highlighted the specific advantages of robotic technology over conventional laparoscopy. This tailored approach reinforces the role of RLR as the preferred modality for complex hepatobiliary surgery.

Nevertheless, although the superiority of RLR over LLR remains a topic of ongoing debate, the international consensus conferences on robotic surgery held in 2018 and 2023 provided clear guidance on its application [10], reaffirming the potential advantages of RLR, including reduced blood loss, shorter hospital stays, lower complication rates, and decreased postoperative pain. A recent multicenter study conducted by Görgec et al. [20] reported that RLR resulted in lower intraoperative blood loss, fewer conversions, and reduced morbidity compared to LLR, but had slightly higher readmission rates. Other studies suggest that RLR may be particularly beneficial in frail or elderly patients [21], who may gain from less physiologic stress, shorter recovery times, and lower complication burdens [22].

Moreover, Balzano et al. [23] demonstrated that, in patients with compensated cirrhosis undergoing minor resections for solitary tumors < 5 cm, RLR was associated with faster procedures, less blood loss, shorter hilar clamping times, and shorter hospital stays compared to LLR. These findings underscore RLR’s potential to match or exceed laparoscopic outcomes in selected patient populations.

Technological advantages of RLR include high-definition 3D imaging, tremor elimination, and enhanced dexterity through articulated instruments such as EndoWrist^®^ devices. These features facilitate delicate dissections near vascular or biliary structures, especially in challenging anatomic locations, such as posterosuperior segments [4,24].

Despite these advantages, certain barriers continue to limit the broader application of RLR including the lack of tactile feedback, high equipment and maintenance costs, longer operative times, and limited instrument range, which may restrict its applicability in some procedures [25,26,27]. Nonetheless, other perioperative outcomes appear comparable between robotic and laparoscopic methods, and current oncologic evidence suggests no significant differences [10]. However, as evidence supporting its benefits grows, adoption of robotic platforms has increased significantly in recent years. This suggests that while RLR may not universally surpass the LLR, they offer comparable safety and efficacy, even though with higher associated costs [9,28].

It has been suggested that in high-volume centers RLR could be more cost-effective as procedural volume helps distribute the high initial and maintenance costs across numerous cases. Additionally, RLR often allows patients to recover more quickly, enabling a faster return to work and reducing the indirect costs of lost productivity, further enhancing the economic benefits of robotics in such settings [24]. Knitter et al. [29] found no significant difference in total cost between RLR and LLR for major hepatectomies. Despite higher upfront costs, overall expenses appear similar, likely due to comparable operative and postoperative resource utilization.

Major RLR appear to reduce blood loss compared to LLR, improving patient outcomes and potentially decreasing the need for transfusions [25], a particularly valuable benefit in larger, complex resections where precision and control are essential. This reduction in blood loss may be attributed to enhanced visualization and precise control of dissection planes, despite the absence of ultrasonic devices in robotic systems. Additionally, the technology’s ability to stabilize tremors enables more refined movements, which is especially beneficial in complex liver resections involving delicate vascular structures [30,31], making RLR a viable alternative in challenging cases, where its advantages in precision and ergonomics may justify the added expense, even if it does not consistently provide cost savings over laparoscopy [4].

Further studies are needed to solidify the long-term advantages and limitations of RLR, particularly in terms of cost-effectiveness and applicability across different clinical settings.

In our experience, both low- and intermediate-difficulty resections (Iwate 1–3 and 4–6) were performed from the beginning of the robotic program. Low-difficulty cases were the most frequent in the early phase, then we observed a shift towards more difficult cases likely because simpler resections were often performed laparoscopically, reserving robotic surgery for more technically demanding cases.

The senior surgeon’s long-standing experience in complex hepatobiliary surgery was instrumental in the safe introduction of the robotic program. His expertise was crucial not only in patient selection and intraoperative decision-making but also in developing standardized procedural steps and in training the surgical team, thereby ensuring a structured and reproducible implementation of the robotic approach. A learning curve was evident for low-difficulty resections, with a progressive reduction in console time over the initial cases. This likely reflects increased confidence and technical refinement with the robotic platform.

Conversely, operative times for intermediate-difficulty cases remained relatively stable, possibly because increasing anatomical and procedural complexity offset the benefits of technical proficiency as the program matured.

In our series, the overall morbidity rate was 24%, with severe complications (Clavien–Dindo grade III–IV) observed in 10% of cases. These rates are comparable to those reported in other studies [32]. Biliary leaks occurred in approximately 6% of cases, ascites in 3.3%, hepatic failure in 0.5% (1 case), and overall mortality was 1.3%. The reoperation rate was 5%. These outcomes are consistent with current literature [8,33], reinforcing the safety and reliability of our approach and aligning with established benchmarks for minimally invasive liver surgery.

The median length of hospital stay was 5 days, reflecting the beneficial impact of our existing ERAS protocol. Nonetheless, there is potential for further improvement by incorporating additional elements such as expanded prehabilitation strategies and more standardized postoperative care pathways. These enhancements could further reduce length of stay and optimize recovery outcomes.

We observed a low rate of Pringle maneuver application, limited to only 3% of cases, without a significant increase in intraoperative blood loss, which averaged 159 mL. This represents one of the main findings of our study. It highlights that, even without subjecting the hepatic parenchyma to temporary vascular deprivation, the robotic approach allows for a more precise dissection and preparation of both small and major vascular pedicles for secure control. This is further supported by the very limited intraoperative blood loss observed in our series. This finding may be attributed to the precision, enhanced visualization, and instrument stability offered by the robotic platform. Moreover, the predominance of minor resections in our series likely contributed to the reduced need for vascular inflow control. Parenchymal transection was performed using the Kellyclasia technique with bipolar and monopolar cautery, which rendered the use of additional devices such as ultrasonic dissectors unnecessary. In our series, the use of the Pringle maneuver was overall very low (3% of resections). This percentage is mainly influenced by the high proportion of minor resections (91%), in which vascular control is rarely required. However, when the analysis is restricted to major resections only, the rate of Pringle maneuver utilization rises to approximately 39%, which is consistent with the rates reported in the literature (25–30%) [5].

Finally, an interesting emerging aspect in this field is the accelerated learning curve associated with RLR, which represents a notable advantage over traditional approaches. Evidence suggests that robotic surgery requires fewer cases to achieve proficiency compared to conventional laparoscopy, primarily due to the ergonomic design, intuitive instrument control, and enhanced three-dimensional visualization offered by robotic platforms [34]. However, it is important to acknowledge that most surgeons have transitioned to robotic surgery only after acquiring substantial laparoscopic experience. The implementation of structured training programs has further contributed to shortening the learning curve, facilitating the broader adoption of robotic techniques, even for major liver resections [35].

This study has several limitations. First, its retrospective and single-center design may introduce selection bias and limit the generalizability of the results. Second, the cohort was heterogeneous, including patients with different histologies, liver function statuses, and preoperative treatments, which may have influenced perioperative outcomes. In particular, the complexity of surgical procedures in patients with cirrhosis is inherently higher compared with those without underlying chronic liver disease. Similarly, patients with colorectal cancer liver metastases often receive preoperative chemotherapy, which may induce varying degrees of liver injury or steatosis, potentially affecting surgical performance and postoperative recovery. These intrinsic limitations, related to patient heterogeneity, should be taken into account when interpreting our results. Third, the majority of procedures were minor resections, which could underestimate the actual risks and challenges associated with major robotic hepatectomies. Moreover, all procedures were performed under the guidance of a single highly experienced hepatobiliary surgeon, which may limit the reproducibility of these results in centers with less surgical expertise. The lack of a direct comparison between robotic liver resection and laparoscopic liver resection represents another limitation of the present study; however, such a comparison was not the focus of our analysis, which was specifically dedicated to robotic surgery. This issue has already been extensively addressed in the international literature, and an additional comparison in our cohort would not have provided novel insights. Finally, long-term oncological outcomes were not assessed and remain to be evaluated in future studies.

RLR represents a significant advancement in MILS, offering a viable alternative to traditional laparoscopic approach. Our 10-year experience demonstrates that RLR can be safely and effectively implemented in a medium-volume HPB center, yielding perioperative outcomes that align with those reported by high-volume institutions [9,33]. This case series confirms the feasibility and safety of RLR across a broad spectrum of hepatic resections. As robotic technology and surgical expertise continue to evolve, RLR is expected to play an increasingly central role in hepatobiliary surgery.

## 5. Conclusions

Hepatobiliary surgery is undergoing constant refinement, and the introduction of robotic technology in recent years has helped overcome many of the technical limitations associated with other surgical approaches. Through a structured robotic hepatobiliary program and with adequate surgical expertise, these procedures can be performed safely even in medium-volume centers, ensuring excellent outcomes in appropriately selected patients. As experience grows and current limitations are progressively addressed, the numerous advantages offered by the robotic approach are likely to support its increasingly widespread adoption in this field.

## Figures and Tables

**Table 1:** Patients demographics (*N* = 150).

Variable	Value
Age > 70	60 (40%)
Sex	
Male	100 (66%)
BMI (kg/m^2^)	26 ± 4 (range 21–49)
ASA score	
I–II	102 (68%)
III–IV	48 (32%)
Previous abdominal surgery	57 (38%)
**Comorbidities**	
Diabetes	22 (15%)
Hypertension	54 (36%)
Cardiovascular disease	37 (25%)
Pulmonary disease	15 (10%)
Cirrhosis	74 (49.3%)
**Tumor indication**	
Hepatocellular carcinoma (HCC)	72 (48%)
Colorectal liver metastases (CRLM)	31 (21%)
Cholangiocarcinoma (CCA)	6 (4%)
Gallbladder carcinoma (GBC)	2 (1%)
Non-CRLM metastases (NCRLM)	13 (9%)
Benign disease	26 (17%)
Lesion characteristics	
Lesion size (mm)	34 ± 25 (range 10–150)
Number of lesions	
Single	124 (83%)
Multiple	26 (17%)
Preoperative chemotherapy	40 (26%)
R0 resection achieved	123 (82%)
Tumor markers	
AFP (*n* = 72)	Median 5.9 (range 0–15,384)
CEA (*n* = 32)	Median 1.5 (range 0–28.5)
CA 19.9 (*n* = 33)	Median 18.7 (range 0–592)
**Type of resection**	
Wedge resection	93 (62%)
Anatomical segmentectomy	23 (15.5%)
Bisegmentectomy	21 (14%)
Left hepatectomy	8 (5%)
Right hepatectomy	4 (3%)
Central hepatectomy	1 (0.5%)
**Iwate difficulty score**	
Low (1–3)	57 (38%)
Intermediate (4–6)	75 (50%)
Advanced (7–9)	11 (7%)
Expert (10–12)	7 (5%)

**Table 2:** Cirrhotic patients (*n* = 74).

Variable	Value
**Etiology**	
Alcohol-related cirrhosis (ALC)	31 (42%)
Non-alcoholic steatohepatitis (NASH)	18 (24%)
Hepatitis C virus (HCV)	16 (22%)
Hepatitis B virus (HBV)	5 (7%)
Other causes	4 (5%)
Portal hypertension	27 (36%)
Child–Pugh class	
A	67 (91%)
B	7 (9%)
Platelet count (×10^3^/µL)	188 ± 80 (range 80–415)
MELD score	9 (range 6–18)

**Table 3:** Secondary lesions (*n* = 44).

Secondary Lesions	Value
CRLM	31 (70%)
GIST	3 (7%)
NET	2 (4.5%)
Melanoma liver metastasis	3 (7%)
Ampullary Adenocarcinoma, (AAC)	1 (2.3%)
Adenoidocistic carcinoma, (AdCC)	1 (2.3%)
Thymoma	1 (2.3%)
Endometrial cancer	1 (2.3%)
PDAC	1 (2.3%)

**Table 4:** Operative and postoperative outcomes (*N* = 150).

Variable	Value
Operative outcomes	
Operative time (min)	250 ± 119 (range 77–840)
Console time (min)	184 (range 5–700)
Conversion to open surgery	18 (12%)
Associated procedures	11 (8%)
Drain placement	82 (55%)
Pringle maneuver	5 (3%)
Blood loss (mL)	159 ± 228 (range 0–2000)
Blood loss < 100 mL	99 (66%)
Blood transfusion	8 (5%)
ICG fluorescence used	122 (81%)
Postoperative outcomes	
Length of hospital stay (days)	7 (mean); median 5 (range 2–46)
Overall morbidity	36 (24%)
Clavien–Dindo grade ≥ III	15 (10%)
Hemorrhage	2 (1.3%)
Biliary leakage	9 (6%)
Reoperation	8 (5%)
Ascites	5 (3.3%)
Liver failure	1 (0.5%)
Readmission within 30 days	4 (3%)
Mortality	2 (1.3%)

## Data Availability

The data supporting this study’s findings are available upon request from the corresponding author, due to privacy reasons as well as ethical restrictions, data cannot be available publicly.

## Abbreviations

AFP	Alpha-fetoprotein
ALC	Alcoholic Liver Cirrhosis
ALPSS	Associating Liver Partition and Portal vein ligation for Staged hepatectomy
ASA	American Society of Anaesthesiologists
CA19-9	Carbohydrate Antigen 19-9
CCA	Cholangiocarcinoma
CEA	Carcinoembryonic Antigen
CRLM	Colorectal liver metastases
GBC	Gallbladder cancer
ERAS	Enhanced Recovery After Surgery (ERAS)
GIST	Gastrointestinal stromal tumor
HCC	Hepatocellular carcinoma
ICG	Indocyanine Green
LLR	Laparoscopic liver resection
LOS	length of stay
MELD	Model for end-stage liver disease
MILS	Minimally invasive liver surgery
NASH	Non alcolic steat hepatitis
NCRLM	Non colorectal liver metastases
PDAC	Pancreatic ductal adenocarcinoma
RLR	Robotic liver resection
